# Systematic Review Shows Only Few Reliable Studies of Physical Activity Intervention in Adolescents

**DOI:** 10.1155/2014/206478

**Published:** 2014-07-20

**Authors:** Nara Michelle Moura Soares, Arley Santos Leão, Josivan Rosa Santos, Glauber Rocha Monteiro, Jorge Rollemberg dos Santos, Sara Maria Thomazzi, Roberto Jerônimo dos Santos Silva

**Affiliations:** ^1^Postgraduate Program in Health Sciences, Federal University of Sergipe, São Cristóvão, SE, Brazil; ^2^Postgraduate Program in Physical Education, Federal University of Sergipe, Avenida Marechal Rondon s/n, Jd. Rosa Elze, São Cristóvão, SE, Brazil; ^3^Department of Education, SE, Brazil

## Abstract

*Introduction*. Several studies have pointed to the high prevalence of low levels of physical activity in adolescents, suggesting the need for more effective interventions for this group. The aim of this study was to present evidence of intervention programs for efficacy of physical activity for adolescents. *Methods*. Surveys in PubMed, SportDiscus, LiLacs, and SciELO databases were conducted using keywords to identify population, intervention, and outcome, as well as DeCS and MeSH terms in English, Portuguese, and Spanish, whenever appropriate. The review included observational studies with minimal intervention of six months, minimum sample size of 100 adolescents, written in any language, and those who have reached STROBE score greater than 70%. *Results*. Only seven studies met all inclusion criteria. Of these, five were pre- and postintervention and two had *n* > 2000 participants. Interventions were of several types, durations, and strategies for physical activity implementation. Behavior change was assessed in 43% of studies and three reported success in some way. *Conclusion*. Due to heterogeneity in their contents and methodologies, as well as the lack of jobs that accompany adolescents after the intervention period, one cannot draw conclusions about the actual effects of the intervention programs of physical activity on the behavior of young people.

## 1. Introduction

Low levels of physical activity in children and adolescents are increasingly presented as a public health problem because of its direct relationship with obesity and other chronic degenerative diseases [1, 2]. Currently, research has shown high levels of physical inactivity among children and adolescents in several countries: Brazil 50–70% [3], U.S. 65–82%, Spain 80–87%, and Russian 85–92% [4], both sexes (male and female, resp.).

Hardy et al. [5] observed sedentary behavior among Australian girls at the beginning and during adolescence and found that there was a 63% increase in the adoption of sedentary behavior during this period.

These same studies suggest interventions for this population, occurring in the community, school environment, and the day-to-day life of children and adolescents so that they are instrumental to the adoption of a physically active behavior, especially by strong and well-documented relationship between physical inactivity and certain chronic diseases [3, 4, 6].

There is a decrease between 60 and 70% levels of physical activity from childhood to adolescence [6, 7] and the explanation for this aspect is not well described in literature. However, all studies agree on the need for interventions to improve and increase levels of physical activity among these youths, preventing its decrease with increasing age.

Accordingly, Twisk [8] reported that the practice of physical activity for children and adolescents promotes healthy behavior in adulthood. Likewise, Tassitano et al. [6] suggest that the early exposure to physical inactivity makes such behavior more difficult to be modified in adulthood and suggest that intervention that meet the specific needs of young people in order to improve this framework programs is developed.

In this sense, the literature shows that there are several studies on physical activity interventions in school environments and out of school, but most of them are applied for periods of up to six months, which sometimes leads to biased interpretations by not considering a long intervention period.

Thus, the aim of this systematic review was to present evidence of intervention programs for efficacy of physical activity for adolescents.

## 2. Methods

### 2.1. Literature Search Strategy

The systematic review of primary studies was carried out in the following databases: MEDLINE, SportDiscus, Virtual Health Library, and SciELO, without limit starting date and using the month of May 2013 as final cutoff.

A search strategy for MEDLINE was developed and accessed via PubMed, based on medical subject headings (MeSH) that were identified as keywords and terms of articles. For LiLacs, descriptors in Health Sciences (DeCS) were use. The reference lists of all articles included were reviewed.

The strategy for SportDiscus used the DeCS terms and their synonyms to identify articles. However, the strategy adopted for SciELO used keywords to identify a larger number of articles.

Various combinations of keywords “adolescents,” “health,” “physical activity,” and “intervention studies” have been used in English, Portuguese, and Spanish, whenever appropriate, identifying “adolescence,” “adolescence health,” “motor activity,” “health promotion,” “health behavior,” “school health,” “life style,” and “intervention studies” crossed with Boolean connectors AND, OR, or AND OR.

The search strategy was based on the identification of the population of adolescents, physical activity intervention, and health-related outcomes, plus the filter by intervention studies.

### 2.2. Inclusion and Exclusion Criteria

The following inclusion criteria were adopted, which were applied independently: (a) cross-sectional, case-control, cohort, and intervention studies (including randomized and quasiexperimental), (b) studies with adolescents (which according to the MeSH and DeCS concept is characterized by age range from 13 to 18 years) or who performed analysis in a subgroup of participants at this age group, (c) studies that had follow-up for at least six months of intervention, (d) studies with a minimum sample size of 100 individuals, (e) studies carried out in school environments, home, community organizations, or interventions at environmental level, and (f) studies written in any language.

Interest interventions were those that presented a change in the physical activity behavior as outcome. The study analyzed the differences found between intervention and control groups related to physical activity, physical fitness, body composition, bone components, psychosocial constructs, and sedentary activities.

Studies carried out with overweight or obese children or adolescents or with those with specific diseases such as chronic diseases were excluded. The review also excluded systematic reviews, narrative reviews, overviews and meta-analyses, and studies with animal models.

### 2.3. Data Extraction

It is noteworthy that the agreement between reviewers was measured by the Kappa index. Two independent reviewers made the search for articles in databases, who analyzed titles, and abstracts and reviewed full papers in order to assess their eligibility for inclusion in the study. All disagreements were discussed and, if they remained, a third reviewer was consulted.

Standardized spreadsheets were created for data extraction according to STROBE guidelines (strengthening the reporting of observational studies in epidemiology statement) so that reviewers could extract information about the characteristics of each study, participants, eligibility criteria, intervention methods, and observed outcomes, including variability measures whenever available.

In data extraction, outcomes related to physical activity (e.g., maximal oxygen uptake, psychosocial variables, and nutritional status) were considered as results. Each article received a double review by researchers to extract this information.

### 2.4. Quality Assessment of Individual Studies

The aim of this step was to use a judicious approach based on high-quality original works to develop evidence levels on physical activity for adolescents that could be used to formulate recommendations for the guidance of health promotion programs.

To assess the methodological quality and inclusion and exclusion criteria, the STROBE guidelines were used as a reference. The articles were read and distributed into three categories: “A” (articles that obtained scores ≥70% of the criteria requested, featuring low risk of bias), “B” (those who met from 50 to 69.9% of the criteria requested, featuring moderate risk of bias), and “C” (articles that met less than 50% of the criteria, featuring with high risk of bias).

## 3. Results

### 3.1. Inclusion of Studies

**Figure 1:**
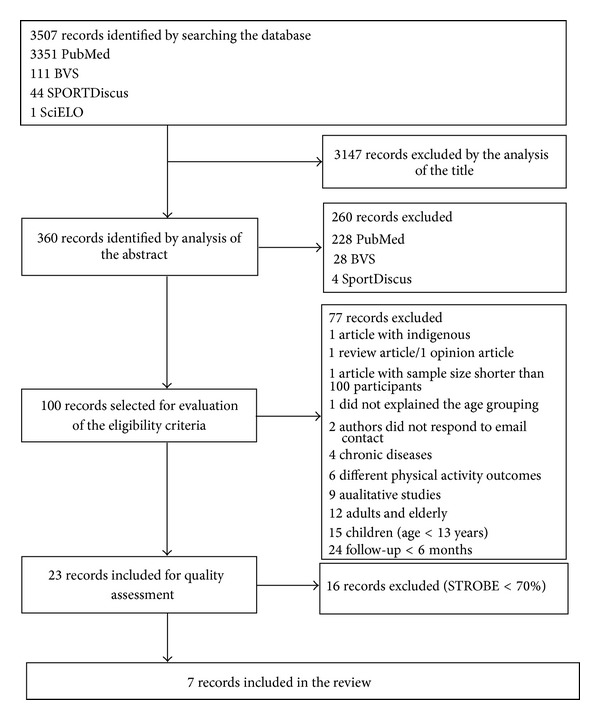
Flowchart of the selection of studies through the inclusion criteria.

After searching databases, 3507 articles were found. Of these, 3147 were excluded from the evaluation of the title, leaving 360 articles identified as eligible for the analysis of the abstract, but 260 were excluded based on the abstract. Of the 100 selected articles, 23 were selected for reading the full text and 16 articles were excluded due to the high risk of bias (Figure 1).

At the end of the research process, seven studies met the inclusion criteria. Interventions were alphabetically arranged by the surname of the first author (Table 1).

### 3.2. Design and Sample

Of the seven studies included, there was only one cohort with only one pre- and postintervention group. The six other studies had experimental design, namely, with the formation of two groups and randomization of subjects for their allocation. All articles showed intervention performed outside the school curriculum.

This work was limited to interventions presenting sample with 100 participants or over, and only two studies [12, 13] showed *n* > 2000. The age of participants ranged from 9 to 15 years and all interventions were performed for individuals of both sexes.

### 3.3. Intervention

Interventions showed varying duration: 6 months [9, 10, 14], 12 months [15], 24 months [12], 36 months [11], and 66 months [13]. All studies used the school environment only for sample recruitment.

The studies also showed varied intervention types. Three articles dealt with physical activity interventions associated with diet/nutrition [9, 14, 15], one study examined the psychosocial dimension of physical activity [12], two assessed physical activity as a primary outcome [10, 11], and one approached behavior change to improve health [13].

All studies presented various intervention strategies with implementation of physical activity [10], game/questionnaire [9], parental training [11], psychosocial scale effect [12], text message [14], conversion of the questionnaire score into METs [15], and social marketing [13].

### 3.4. Outcome and Intervention Measure

In 3/7 of the studies (*n* = 3), the change in behavior related to physical activity was measured through parents' guidance about the importance of physical exercise [11], questionnaire [12], and pedometer [14].

Some articles did not include behavior change as its main objective, but information was entered as a result of actions. These studies used measures such as body mass index [9], participation frequency [10], physical activity recall [15], and encouragement for physical activity [13]. It is important to report that only 43% (*n* = 3) of interventions reported success in some way (e.g., change in the practice of physical activities, dietary intake, and psychosocial scale).

### 3.5. Quality of Studies

In the individual assessment of the quality of studies, there was 100% agreement in the analysis of titles (*k* = 1.00, *P* < 0.01, 95% CI 1.00 to 0.897) and 89.9% in the analysis of abstracts (*k* = 0.899, *P* < 0.01, 95% CI 1.00 to 0.703). According to the STROBE checklist, there were some critical points in the analysis of the quality of studies, so that after initial and individual quality evaluation, study had score less than 50%; 15 studies had scores from 50 to 69.9% and seven studies had score equal to or greater than 70%.

Only 40.7% of articles indicated the study design with term commonly used in the title or abstract, 37% explained how they calculated the sample size, 22.2% explained how missing data were treated, 29.6% gave reasons for the nonparticipation of subjects in each phase of the study, 25.9% assessed the relevance of presenting a flow diagram, 11.1% indicated the number of participants with missing data for each variable, and 33.3% reported the source of funding and the role of sponsors in the study.

## 4. Discussion

### 4.1. Intervention

The purpose of this systematic review was to summarize and present evidence related to intervention programs for efficacy on physical activity for youth. Based on the seven interventions resulting from the qualitative assessment, there is evidence of the need for more accurate studies or with better design for this type of intervention within the school environment, because the teen spends most of its time in this place and these needs to be optimized.

This work was limited to articles with expressive sample size and long follow-up period conditions that directly influence behavior change, verifying that this ranges from six to 66 months.

The World Health Organization [16] recommends that children and adolescents should perform 60 minutes of moderate to vigorous physical activity per day, and these activities should involve aerobic exercise, muscle strengthening, and bone strengthening. The interventions analyzed in this study did not specify type, frequency, and duration of physical activity sessions.

Five studies used different approaches to measure the expected intervention result [9–13]. Lubans et al. [14] and Rosenberg et al. [15] presented similar measurements to quantify the intervention success. Lubans et al. [14] used pedometer and Rosenberg et al. [15] used accelerometer to quantify physical activity levels.

Likewise, the meta-analysis by Metcalf et al. [17] reported results of studies with 30 interventions to promote physical activity in children and adolescents. There was a comparison between the intervention and the amount of physical activity performed during the study, with no significant change in the total time spent in physical activities, with small improvements in the time spent in activities from moderate to vigorous intensity (about four minutes per day) and intervention performed at home or in the school environment. This finding may explain at least in part why these interventions have had limited success in reducing BMI or body fat in children. It is believed that interventions using text messages are effective strategies that stimulate young people to perform physical activity. Also, another strategy also proved effective in this study was the inclusion of parents in modifying unhealthy habits process.

Grydeland et al. [18] conducted a randomized controlled trial to assess the effect of physical activity intervention lasting 20 months in 700 adolescents and found a trend of improvement for the intervention group, and this effect is more evident in girls and among participants with low physical activity levels compared with boys and with participants with high physical activity levels, respectively. Apparently, the intervention programs of physical activity have proven to be effective for individuals who have low levels of physical activity and among females; however, none of the studies found similarity which is found here with this finding. It is not clear how the behavior of this relationship, since persistence in programs, may cause a disincentive of those participants in the progression of activity.

The results of this review show that the school environment was used only for sample recruitment and not as basis or reference to intervention of PE curriculum component.

It is recommended that physical education is used as an important strategy in promoting healthy habits [19–21]. de Meester et al. [22] conducted a systematic review and observed that interventions performed in the school environment among European adolescents led to a short-term improvement in physical activity levels. This review found no medium- and long-term studies that combined show evidence of physical education programs performed in the school to promote changes in the habits of children and adolescents, presenting itself as an important gap to be filled by studies that intend to identify effective intervention results of physical activity in adolescents.

Several strategies have been used to increase the physical activity levels among children and adolescents. This study verified the implementation of supervised training, the use of game or questionnaire, guidance to parents about the importance of physical exercises, psychosocial scale effect, text messaging on mobile, conversion of questionnaire scores into METs, and social marketing strategy.

However, the studies that were successful were what used to instruct parents about the importance of exercise [11], use of psychosocial shares questionnaire [12], and the use of pedometer [14], which indicates a path to be followed in the systematization of physical activity interventions for the methods to be used indicating that subjective, direct, and external to the subject methods can be used for this purpose.

The studies included in this review showed effective interventions in promoting an active lifestyle outside the school, but it is believed that if the activities were associated with the physical education curriculum component, the effect would be more comprehensive.

A systematic review carried out by Atkin et al. [23] to identify interventions aiming to promote physical activity in adolescents held after school has concluded that these activities were effective in the short-term, suggesting that short-term interventions, when meeting the minimum criteria required for the age group analyzed, are more effective in the period after school.

Dobbins et al. [24] conducted a meta-analysis of 44 studies with 36,593 participants and verified the effectiveness of school-based physical activity intervention in the promotion of health and fitness in children and adolescents. The authors observed a positive impact of the involvement of participants in moderate to vigorous activities but with no progression in the physical activity levels.

Despite the different approaches in terms of population sample and compared the obtained results presented in this systematic review, can be highlight as strategies for effective participation in physical activity intervention programs as tools, programs drawn up in accordance with the popularity of activities and evaluation of the frequency of participation, inclusion of parents in the programs, frequent evaluation of the intervention of the dose-response to exercise and use text messaging to encourage youth participation. It is observed that physical activities performed outside school are an important tool to encourage behavior change, since interventions bring positive effects on health-related outcomes; however, it is believed that interventions should be carried out within the school context embedded within the curriculum of physical education, but there is no clear studies that show how these interventions may accept the claims of the WHO with regard to frequency, duration, and intensity of exercise.

Studies indicate that an intervention requires a clear outcome, so that an intervention with adolescents should have the intention to change behavior regarding regular physical activity and other associated behaviors, so there is the need to follow up after the period of proposed intervention, in order to determine whether there was a real change of behavior in the subject.

### 4.2. Quality of Studies

The use of observational studies lasting at least six months and minimum sample size of 100 participants are among the inclusion criteria of this systematic review. We believe that experimental studies with this intervention period and sample size are scarce in literature, prioritizing quality evaluation by means of the STROBE guidelines.

However, the studies included in this review showed high quality, fulfilling the checklist recommendations, and only one study used pre- and posttest design and the others included a control group for comparison purposes.

Another important aspect is related to the sample size, as it is related to the size of the treatment effect, improving the power of analysis of studies included in this review. Despite the high quality of studies, the true cause and effect relations cannot be analyzed due to various forms of interventions that caused improvements in the treatment group.

## 5. Conclusion

This study shows that a small number of high quality items were successfully related to the intervention of physical activity in adolescent programs. Due to heterogeneity in their contents and methodologies, as well as the lack of jobs that accompany adolescents after the intervention period, one cannot draw conclusions about the actual effects of the intervention programs of physical activity on the behavior of young people.

However, this study points to the need for change on two fundamental elements: (a) academic, with more elaborate studies with greater scientific rigor so that this information will reflect reality and (b) intervention, indicating that there is need for change in the curriculum of physical education and physical activity for adolescents (as we expected that physical education would supply this demand) or as an extracurricular component.

The results show that for an intervention to be effective physical activity needs to be structured with the support and understanding of parents or guardians, having the primary outcome as exercise and use of direct measures for the verification of the increased levels of physical activity.

It is believed that it is necessary for medium- and long-term intervention programs of physical activity in the school environment, especially considering that the school is the best place for the behavior of regular practice of physical activities to be acquired. Moreover, if activities are related to the physical education curricular component, the effect could be much more comprehensive.

## Figures and Tables

**Table 1:** Summary of characteristics and results of studies with STROBE > 70%.

Study	Sample	Design	Measure	Intervention program	Outcomes
Amaro et al. [9]	*n* = 291 11 to 14 years Both sexes	Experimental—two groups: randomization and intervention and control group Nonschool physical activity	BMI Kalèdo questionnaire to assess knowledge about nutrition (31 items), physical Activity (eight items), food intake (34 items)	Six-month follow-up Subjects played only once for 15 to 30 min and after six months were reevaluated BMI was assessed for all subjects	There was no significant difference in average daily time spent in physical activities between intervention (2.1 h, 95% CI 1.9 to 2.3) and control groups (2.2 h, 95% CI 2.0 to 2.4) Knowledge about nutrition showed no difference between intervention and control groups (*P* < 0.05) Knowledge about food intake showed no difference between intervention and control groups (*P* < 0.01)

Bush et al. [10]	*n* = 221 11 to 16 years Both sexes	Experimental—two groups: randomization and intervention and control group Nonschool physical activity	Physical activity leisure: satisfaction in physical activity, frequency of participation in program	Six-month follow-up (a) First 16 weeks—fun activities (b) Half of the implementation phase—program changes according to the popularity of activities and research answers	No increased leisure physical activity and satisfaction in physical activity in the intervention group Participation in the program: 63% for boys and 78% for girls

Hovell et al. [11]	*n* = 117 10 to 13 years Both sexes	Experimental—two groups: randomization and Intervention and Control group Nonschool physical activity	Demographics—ethnicity, parental education: diet—24-hour recall physical activity—24-hour recall DXA-BMD and body composition weight height	36-month follow-up Each session lasted 3 months Procedure of parental training—90 minutes of instruction for 8 weeks Procedure of children training—90 min of instruction for 8 weeks with 60 min of physical activity	Intervention increased 25% calcium intake; Intervention attenuated the decline in high-impact physical activity

Huhman et al. [12]	*n* = 2257 9 to 13 years Both sexes	A pre-/postintervention cohort group Nonschool physical activity	YMCLS: (a) nonschool physical activity; (b) physical activity in the last 7 days; (c) physical activity the day before intervention	Psychosocial dimensions of physical activity followed for 24 months. (a) Expectations: beliefs about the benefits of participating in physical activities. (b) Self-efficacy: confidence to overcome obstacles in the practice of physical activity. (c) Social influences: the influence of family and colleagues.	Dose-response effect: (a) exercise—the day before intervention; (b) average number of sessions; (c) during spare time positive effect on psychosocial scales, social influences, and self-efficacy

Jago et al. [13]	*n* = 4063 11 to 13 years Both sexes	Experimental—two groups: randomization and intervention and control group Nonschool physical activity	Nonschool physical activity, nutrition	66-month follow-up (a) Complete change of school meals; (b) Change in behavior to improve health; (c) Social marketing to increase water consumption, encourage physical activity, differentiate food qualities, and energy balance	Two thirds of boys and one third of girls showed low fitness levels at baseline Time of moderate to vigorous physical activity at baseline: 96 min for control and 103 min for intervention There was no statistical difference in fitness and levels of moderate to vigorous physical activity

Lubans et al. [14]	*n* = 106 14.1 (±0.8) years Both sexes	Experimental—two groups: randomization and intervention and control group Nonschool physical activity	Physical activity—pedometer SPANS—sedentary behavior and diet	6-month follow-up Once a week, the content of school sports was presented Daily sending of text message to increase health levels	The intervention group increased step counting/days compared to the control group (956 to 4107 steps/day—boys; and 999 to 1999 for girls) The intervention group had no significant effect on any sedentary behavior

Rosenberg et al. [15]	*n* = 878 10 to 15 years Both sexes	Experimental—two groups: randomization and intervention and control group Nonschool physical activity	PAR: accelerometer, time spent in sedentary activity, demographics	12-month follow-up The result of the physical activity recall was converted into METs The covariation of the change in diet, physical activity, and sedentary behavior was examined	No covariation was found between physical activity and sedentary behavior, physical activity and diet, or diet and sedentary behavior

YMCLS: youth media campaign longitudinal survey; SPANS: schools physical activity and nutrition study.
